# Application of PCR amplicon sequencing using a single primer pair in PCR amplification to assess variations in *Helicobacter pylori *CagA EPIYA tyrosine phosphorylation motifs

**DOI:** 10.1186/1756-0500-3-35

**Published:** 2010-02-10

**Authors:** Hans-Jürg Monstein, Anneli Karlsson, Anna Ryberg, Kurt Borch

**Affiliations:** 1 Clinical Microbiology, Molecular Biology Laboratory, University Hospital, S-581 85 Linköping, Sweden; 2 Division of Surgery, University Hospital, S-581 85 Linköping, Sweden; 3 Department of Clinical and Experimental Medicine, Faculty of Health Sciences, Linköping University, S-581 85 Linköping, Sweden

## Abstract

**Background:**

The presence of various EPIYA tyrosine phosphorylation motifs in the CagA protein of *Helicobacter pylori *has been suggested to contribute to pathogenesis in adults. In this study, a unique PCR assay and sequencing strategy was developed to establish the number and variation of *cagA *EPIYA motifs.

**Findings:**

MDA-DNA derived from gastric biopsy specimens from eleven subjects with gastritis was used with M13- and T7-sequence-tagged primers for amplification of the *cagA *EPIYA motif region. Automated capillary electrophoresis using a high resolution kit and amplicon sequencing confirmed variations in the *cagA *EPIYA motif region. In nine cases, sequencing revealed the presence of AB, ABC, or ABCC (Western type) *cagA *EPIYA motif, respectively. In two cases, double *cagA *EPIYA motifs were detected (ABC/ABCC or ABC/AB), indicating the presence of two *H. pylori *strains in the same biopsy.

**Conclusion:**

Automated capillary electrophoresis and Amplicon sequencing using a single, M13- and T7-sequence-tagged primer pair in PCR amplification enabled a rapid molecular typing of *cagA *EPIYA motifs. Moreover, the techniques described allowed for a rapid detection of mixed *H. pylori *strains present in the same biopsy specimen.

## Background

*Helicobacter pylori *is a microaerophilic Gram-negative bacterium that chronically infects the gastric mucosa. It is recognised as a human pathogen associated not only with chronic gastritis [1], but also with peptic ulcer [2] and gastric cancer [3]. A commonly used molecular marker of *H. pylori *virulence is the *cagA *gene (cytotoxin-associated gene) [4], which is a part of the 40 kb Cag-Pathogenicity Island (*cag*-PAI) [5]. The CagA cytotoxin is directly injected into epithelial cells by a type IV secretion system, encoded by genes located in the *cag*-PAI [6-8]. In the host cell, CagA localises to the plasma membrane and undergoes phosphorylation on specific tyrosine residues within repeating penta amino acid Glu-Pro-Ile-Tyr-Ala (EPIYA) motifs, present at the C-terminus of the protein [9,10]. The C-terminal part, which contains the EPIYA motifs, has been shown to be highly variable, as opposed to the highly conserved N-terminal part [7,11-13]. CagA EPIYA motifs are defined as EPIYA-A, -B, -C, and -D, according to the amino acid sequences that surround the EPIYA sequence [10,13,14]. CagA proteins nearly always possess EPIYA-A and EPIYA-B sites, followed by one to three repeats of EPIYA-C in Western-type [13] or EPIYA-D sites in East Asian-type of *H. pylori *clinical isolates [14]. It has been suggested that the variation in number of repeating EPIYA-C or -D motifs determines the biological activity of CagA in phosphorylation-dependent as well as phosphorylation-independent ways [10,15]. It has also been shown that the number of CagA EPIYA-C motifs is an important factor for cancer risk among Western strains [16].

Numerous PCR assays have been reported for the identification of CagA EPIYA phosphorylation motifs [12-14,17,18]. To simplify the determination of the number and types of *cagA *EPIYA motifs present, Argent and co-workers [17] developed an elegant PCR-based approach for identification of individual EPIYA motifs, using a single forward primer and multiple reverse primers. In most studies, *cagA *EPIYA amplicons have been visualised by agarose gel electrophoresis and sequenced using various region specific primers [12-14,17,18].

In this study, we report on the analysis of amplicons derived from a single primer pair by automated capillary electrophoresis combined with direct sequencing using universal sequencing primers to assess variations in the *H. pylori cagA *EPIYA motifs. The technique also works in the presence of multiple *H. pylori *strains in the same biopsy specimen.

## Methods

### Study subjects and tissue collection

Eleven individual archival frozen *H. pylori *positive gastritis tissue samples were used in this study. Preparation of multiple displacement amplified DNA (MDA-DNA) derived from DNA isolations and the detection limit of *Helicobacter pylori *MDA-DNA have been described previously [19,20].

### PCR amplification

**Figure 1:**
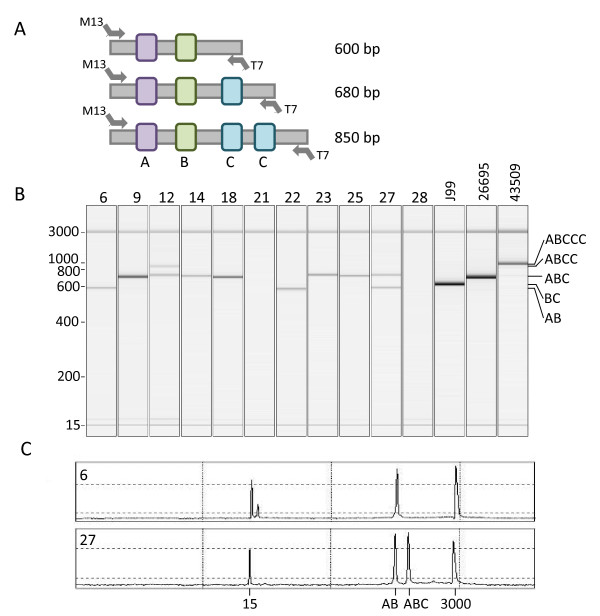
**A) Schematic drawing of the CagA EPIYA motifs detected in our clinical samples and its approximate sizes in bp (including the M13- and T7-sequence tags**. B) Size distribution of the *cagA *EPIYA motif amplicons derived from MDA-DNA of eleven gastritis biopsy specimens (No. 6, 9, 12, 14, 18, 21, 22, 23, 25, 27, and 28). J99, 26695, and 43509 indicate the position of *cagA *EPIYA motif amplicons derived from *H. pylori *J99, 26695, and ATCC 43509^T ^DNA, respectively. A virtual size reference marker is indicated in the left margin and a reference marker for the different EPIYA motif sizes is indicated in the right margin. C) Representative electropherograms revealing the presence of a single (*cagA *EPIYA-AB; No. 6) or two different (*cagA *EPIYA-AB and EPIYA-ABC; No. 27) *cagA *EPIYA motifs, indicating the presence of either one or two isogenic *H. pylori *strains in the same biopsy specimen. 15 and 3000 indicate the position of lower and upper size markers.

The *CagA *gene EPIYA repeat regions were amplified using 10 pmol of each primer M13-CagA-EPIYA.SE (5'-*TGT AAA ACG ACG GCC AGT *CCC TAG TCG GTA ATG GRT TRT CT-3') and T7-CagA-EPIYA.AS (5'-*TAA TAC GAC TCA CTA TAG GG*T GTG GCT GTT AGT AGC GTA ATT GTC-3'), 2 μl of MDA-DNA, and 1× HotStarTaq Master mix (Qiagen, Hilden, Germany) in a final reaction volume of 25 μl. Amplification conditions were as follows: initial denaturation at 95°C for 15 min; 30 cycles of 95°C for 30 s; 55°C for 30 s; 72°C for 1 min; and final extension at 72°C for 10 min. Prior to sequencing, amplicons were analysed by automated capillary electrophoresis using a QIAxcel system and a QIAxcel DNA High Resolution kit (Qiagen, Hilden, Germany), showing amplicons of different sizes depending on the variation and number of repeats (Figure 1). Primers for *CagE *were designed from *H. pylori *strain 26695 [GenBank:AE000511] (*CagE*-M13-sense primer 5'-*TGT AAA ACG ACG GCC AGT *GGG GGA ATA GGT TGT TTG GT-3' and *CagE*-antisense primer 5'-GGA TCA CCC CAT CAT CTA AAA A-3', yielding an amplicon of ~385 bp), whereas *cag*-PAI empty-site primers were from Akopyants and co-workers [6] (M13-sense 5'-*TGT AAA ACG ACG GCC AGT *ACA TTT TGG CTA AAT AAA CRC TG-3' and *cag*-PAI empty-site T7-antisense 5'-*TAA TAC GAC TCA CTA TAG GG*T CAT GCG AGC GGC GAT GTG-3', yielding an amplicon of ~380 bp if the *cag***-**PAI is lost. PCR amplifications were carried out as described above using the following amplification conditions: initial denaturation at 95°C for 15 min; 30 cycles of 95°C for 20 s; 55°C (*cagE*) or 50°C (*cag*-PAI empty-site) for 20 s; 72°C for 40 s; and final extension at 72°C for 10 min. Subsequently, amplicons were analysed by automated capillary electrophoresis as described above.

### DNA sequence analysis

Amplicon sequencing was done with the specified universal primers, via a custom sequencing service (Eurofins MWG Operon, Ebersberg, Germany). The obtained DNA sequences corresponding to *cagA *EPIYA motif repeats, derived from the nine *cagA *positive isolates and three reference strains (*H. pylori *26695, J99, ATCC 43509^T^) were translated into amino acid sequences, aligned and compared with catalogued *H. pylori *26695 [GenBank:AE000511, *H. pylori *J99 [GenBank:AE001439], *H. pylori *P12 [GeneBank:CP001217], *H. pylori *G27 [GenBank:CP001173], and *H. pylori *Shi470 [GeneBank:CP001072] sequences using the CLC DNA Workbench software [21]. Sequences were retrieved from the NCBI nucleotide database [22].

## Results and discussion

We successfully amplified the variable 3'-region of the *cagA *gene in nine of eleven MDA-DNA extracts from *H. pylori *positive gastritis biopsy specimens using a single PCR amplification, followed by automated capillary electrophoresis and universal primer-tagged amplicon sequencing. Electrophoretic analysis of the eleven cases revealed the presence of a single band in seven cases, multiple bands in two cases, while two cases were PCR negative (Figure 1; table 1). The amplicons ranged in size between ~600 and ~900 bp, indicating the presence of varying numbers of *cagA *EPIYA motifs in the different biopsies. Amplicons derived from *H. pylori *26695 and *H. pylori *J99 revealed bands of similar sizes, whereas *H. pylori *ATCC 43509^T ^generated a larger amplicon of ~1000 bp (Figure 1).

**Table 1:** *H. pylori *genotyping

Subject No.	Gastritis classification^a^	16S rDNA^b ^type	*cag*-PAI PCR analysis		CagA EPIYA types
			ES^c^	*cagE*	
6	P-2-na	"Strain A"	-	+	AB
9	A-I-a	J99	-	+	ABC
12	C-3-a	26695	-	+	ABC + ABCC
14	A-I-a	26695	-	+	ABC
18	P-I-na	26695	-	+	ABC
21	P-I-na	J99	+	-	-
22	C-I-a	J99	-	+	AB
23	P-2-na	26695	-	+	ABC
25	P-2-na	26695	-	+	ABC
27	A-I-a	26695	-	+	ABC + AB
28	A-I-na	26695/J99	-	-	-
reference strain		HP 26695	-	+	ABC
reference strain		HP J99	-	+	BC
reference strain		ATCC 43509^T^	-	+	ABCCC

a) Gastritis classification according to the revised Sydney system [24]. **A**: antrum predominant gastritis; **P**: pangastritis; **C**: corpus dominant gastritis; **I**: mild degree; **2**: moderate degree; **3**: severe degree; **a**: atrophy; **na**: no atrophy

b) 16S rDNA variable V3 region motifs established by pyrosequencing analysis [19].

c) ES: *cag*-PAI empty-site, + presence of a 380 bp amplicon, indicating loss of *cag*-PAI; - presence of *cag*-PAI [6].

To assess the presence or loss of *cag*-PAI, *cagE *and *cag*-PAI empty-site PCR assay was carried out. *CagE *was detected in nine of eleven cases corresponding to the results of *cagA *genotyping (Table 1). Amplification of *cag*-PAI empty-site yielded a fragment of ~380 bp in biopsy specimen No. 21, revealing loss of *cag*-PAI. Thus, the result confirms the absence of *cagA *EPIYA motif and *cagE *amplicon in this biopsy specimen. *H. pylori *DNA derived from biopsy specimen No. 28, negative in *cagA *and *cagE *amplification, did not yield any empty-site amplicon of the expected size (Table 1), suggesting the presence of a deviating *cag*-PAI.

To confirm the *cagA *EPIYA motif genotype results obtained by fragment length analysis, we sequenced the amplicons using universal M13- and T7-sequencing primers. In seven of the eleven cases, an AB or ABC (Western type) *cagA *EPIYA motif was present (Table 1). In two additional cases, double *cagA *EPIYA motifs (ABC+ABCC or ABC+AB) were detected. Presumably, this indicates the presence of two individual strains in the same biopsy specimen (Table 1). The analysis of *cagA *EPIYA motifs from mixed *H. pylori *strain infection was possible by a combination of capillary electrophoresis and sequencing. The presence of *cagA *EPIYA-A and EPIYA-B motifs could be determined from the sequencing chromatograms, but the region of the repeating C-motifs contained double peaks caused by amplicons of different sizes and nucleotide compositions. Instead, the high resolution capillary electrophoreses analysis enabled us to determine the number of EPIYA-C motifs by the size of the amplicons.

DNA sequencing of reference strains revealed the presence of a *cagA *EPIYA-ABC motif in *H. pylori *26695, a *cagA *EPIYA-BC motif in *H. pylori *J99, and a *cagA *EPIYA-ABCCC motif in *H. pylori *ATCC 43509^T ^(Table 1).

In previous reports, the 3'-end of the *cagA *gene encoding the EPIYA repeats were analysed by single or multiplex PCR assays and visualisation of amplicons by agarose gel electrophoresis. In most studies, amplicons are sequenced using a battery of gene specific primers (often the PCR primers). DNA sequence analysis of cloned amplicons with universal sequence primers (such as M13 uni -21), targeting sequences flanking cloned inserts [14,16,17], has also been described. The present study describes a unique PCR assay that detects all of the *cagA *phosphorylation sites, including the Asian EPIYA-D type. Tagging of the PCR primers enables rapid sequencing for revealing individual differences in the samples. Moreover, many laboratory workers are also concerned about the use of ethidium-bromide stained agarose gels, which is a health-risk factor. In agreement with a previous study from our laboratory we show that the use of automated capillary electrophoresis, which is a rapid technique that also minimizes the health risk during electrophoresis, overcomes these obstacles [19].

Commonly, work identifying *cagA *genotypes as potential virulence factors has been performed on bacterial isolates cultured from gastric biopsy specimens. However, bacterial culture methods are often time-consuming. In this view, the present and a previous study have shown that direct PCR on MDA-DNA derived from biopsy DNA provides a reliable source for multiple molecular analyses [19]. Using random amplified polymorphic DNA (RAPD) fingerprint analysis, it was found that ~60% of the patients were infected by two or more different *H. pylori *strains [23]. Using the methodological approaches described herein, we were able to detect multiple DNA fragments, indicating that the method indeed is suitable for analyzing mixed *H. pylori *infection in two gastric biopsy specimens (Table 1).

Due to the limited number of biopsies analysed here, we were not able to draw any conclusions regarding a possible correlation between the gastritis classification and *cagA *genotypes. However, the primary goal of the present study was not to perform a clinical study at large but rather to establish a new and simple methodological approach to assess variations in *H. pylori cagA *EPIYA motifs.

Altogether, the single PCR reaction with MDA-DNA as template, in combination with the automated capillary electrophoresis and direct sequencing of universal primer-tagged amplicons, offers a rapid means of genotyping *H. pylori *DNA isolated from biopsy specimens. Moreover, the technique described allowed for a rapid detection of mixed *H. pylori *strains present in the same biopsy specimen.

## Competing interests

The authors declare that they have no competing interests.

## Authors' contributions

HJM, AK, AR and KB participated in the conception, design, drafting of the manuscript, and final approval of the version to be published. HJM, AK and AR were responsible for the acquisition, analysis and interpretation of data. KB collected and selected the biopsy specimens in the study. All authors have read and approved the final manuscript.
